# TSC2/mTORC1 signaling controls Paneth and goblet cell differentiation in the intestinal epithelium

**DOI:** 10.1038/cddis.2014.588

**Published:** 2015-02-05

**Authors:** Y Zhou, P Rychahou, Q Wang, H L Weiss, B M Evers

**Affiliations:** 1 Markey Cancer Center, The University of Kentucky, Lexington, KY, USA; 2 Department of Surgery, The University of Kentucky, Lexington, KY, USA

## Abstract

The intestinal mucosa undergoes a continual process of proliferation, differentiation and apoptosis, which is regulated by multiple signaling pathways. Notch signaling is critical for the control of intestinal stem cell maintenance and differentiation. However, the precise mechanisms involved in the regulation of differentiation are not fully understood. Previously, we have shown that tuberous sclerosis 2 (TSC2) positively regulates the expression of the goblet cell differentiation marker, MUC2, in intestinal cells. Using transgenic mice constitutively expressing a dominant negative TSC2 allele, we observed that TSC2 inactivation increased mTORC1 and Notch activities, and altered differentiation throughout the intestinal epithelium, with a marked decrease in the goblet and Paneth cell lineages. Conversely, treatment of mice with either Notch inhibitor dibenzazepine (DBZ) or mTORC1 inhibitor rapamycin significantly attenuated the reduction of goblet and Paneth cells. Accordingly, knockdown of TSC2 activated, whereas knockdown of mTOR or treatment with rapamycin decreased, the activity of Notch signaling in the intestinal cell line LS174T. Importantly, our findings demonstrate that TSC2/mTORC1 signaling contributes to the maintenance of intestinal epithelium homeostasis by regulating Notch activity.

The intestinal epithelium undergoes a process of constant and rapid renewal. The intestinal crypts of Lieberkühn, a highly dynamic niche with multipotent stem cells residing in its lower third, generate new cells that eventually differentiate into the four specialized cell types of the small intestine, namely absorptive enterocytes and secretory lineages known as enteroendocrine, goblet and Paneth cells.^1, 2^ Differentiated enterocytes, which make up the majority of the cells of the gut mucosa, then undergo a process of apoptosis and are extruded into the lumen.^1, 3^ The mechanisms that regulate stem cell maintenance, proliferation, differentiation and apoptosis must be precisely orchestrated to ensure proper organ maintenance.^3^ An imbalance in this highly-regimented and orderly process within the intestinal crypts is associated with a number of intestinal pathologies, including colorectal cancer, inflammatory bowel disease (IBD) and necrotizing enterocolitis.^4, 5, 6^ To date, the cellular mechanisms regulating intestinal cell differentiation are not entirely known.

Tuberous sclerosis is an autosomal dominant disorder caused by the mutations in the tuberous sclerosis 2 (TSC2) gene.^7^ TSC1 and TSC2 function as a complex and exert their tumor suppressor function by negatively regulating the mTOR pathway.^8^ mTOR is a member of the phosphatidylinositol 3-kinase-related kinase family and regulates protein translation, cell cycle progression and cell proliferation.^9^ The TOR signaling events are essential for epithelial growth, morphogenesis and differentiation in the vertebrate intestine.^10^ mTOR exists in two complexes: mTORC1 (containing mTOR, Raptor etc.) and mTORC2 (containing mTOR, Rictor etc.). REDD1 is proposed to inhibit mTORC1 by displacing TSC2 from the 14-3-3-binding protein, thus allowing TSC2 to inhibit mTORC1.^11^ The bacterially derived drug rapamycin allosterically inhibits mTORC1 activity.^12^

Notch signaling is involved in the control of proliferation, differentiation and development.^13^ Binding of cell surface-tethered ligands (Delta and Jagged) to Notch receptors on neighboring cells initiates a series of cleavages in the Notch receptor. The final cleavage releases the Notch intracellular domain (NICD), which translocates into the nucleus and acts as a transcriptional coactivator that promotes gene expression. Hairy/enhancer of split 1 (Hes1) is one of the best-characterized target genes of the Notch signaling pathway. The Notch-Hes1 pathway promotes the proliferation of intestinal stem/progenitor cells and inhibits secretory cell development.^14, 15, 16^ Hes1 functions as a downstream target of both the Notch and Wnt signaling pathway in LS174T colon cancer cells,^17^ suggesting that crosstalk between Notch and Wnt signaling may take place via Hes1.

Previously, we reported that REDD1/TSC2/mTOR signaling pathway regulates Notch signaling and the expression of mucin2 (MUC2), a goblet cell differentiation marker, in intestinal cell lines.^18, 19^ In our current study, we used transgenic (TG) mice constitutively expressing a dominant negative TSC2 allele, to further elucidate the role of mTOR signaling pathway during the turnover of the intestinal epithelium, including its crosstalk to Notch signaling. We found that TSC2 inactivation increased mTORC1 and Notch activities and disrupted goblet and Paneth cell differentiation. Conversely, treatment of mice with dibenzazepine (DBZ) or rapamycin attenuated the decrease of goblet and Paneth cell generation induced by TSC2 inactivation. Our study demonstrates that TSC2/mTORC1 signaling has an important role in the maintenance of intestinal epithelium homeostasis. Thus, aberrant mTOR signaling may result in an imbalance in the proliferation, differentiation and apoptosis patterns within the intestinal crypts, which is associated with a number of intestinal pathologies.

## Results

### TSC2 is essential for intestinal cell differentiation

Previously, using *in vitro* cell lines, we showed that TSC2 contributes to intestinal goblet cell differentiation.^19^ To further delineate the role of TSC2 in intestinal differentiation, we used TG mice with mutated TSC2. Because TSC2-null mice die *in utero*,^20^ we obtained mice TG for a TSC2 allele lacking Rheb-GTPase-activating protein function (TSC2-RGΔ). This mouse model has been used to study the role of TSC2/mTOR in various types of tissues.^21, 22, 23^

**Figure 1:**
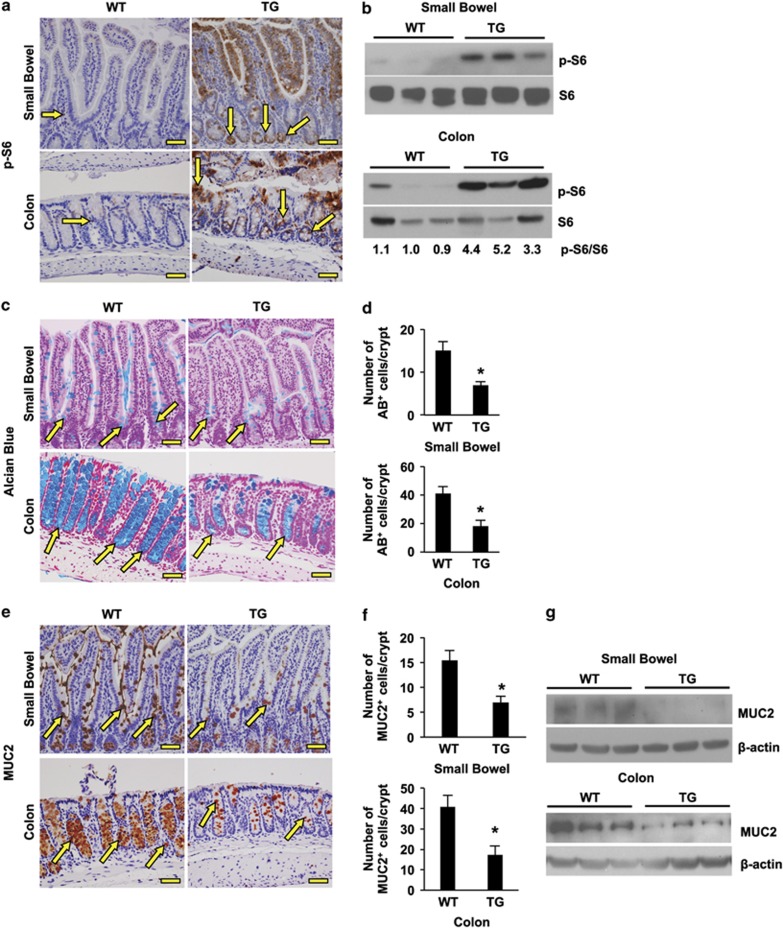
TSC2 inactivation results in altered goblet cell differentiation. (**a**) Immunohistochemical (IHC) staining demonstrated increased expression of phospho-S6 (some are indicated by arrows) in intestinal epithelium of TG mice compared with WT mice. (**b**) Mucosal protein lysates extracted from WT and TG mice were used for western blot detection of phospho-S6 and total S6 protein expression. The levels of p-S6 were quantitated densitometrically and expressed as fold change with respect to total S6. (**c** and **d**) Alcian blue (AB) staining of the intestine revealed a reduction in mucinous goblet cells in TSC2-mutant TG mice compared with WT mice (**c**, some are indicated by arrows). (**d**) Quantification of AB-positive cells in WT- and TSC2-mutant TG mice. (Data represent mean±S.D.; *P<0.05 *versus* WT). (**e** and **f**) (IHC) staining for MUC2 further confirmed the decrease in goblet cells in TSC2-mutant TG mice compared with WT mice (**e**, some are indicated by arrows). (**f**) Quantification of MUC2-positive cells in WT- and TSC2-mutant TG mice. (Data represent mean±S.D.; *P<0.05 *versus* WT). (**g**) Mucosal protein lysates extracted from WT and TG mice were used for western blot detection of MUC2 protein expression. Scale bars, 50 *μ*m

First, we confirmed the presence of the ΔRG transgene by standard PCR using DNA from wild type (WT) and TG mice and primers, as previously described^21, 22, 23^ (Supplementary Figure 1). In TG mice, the intestine appeared normal by histology (Supplementary Figure 2), with no change in apoptotic cell numbers (Supplementary Figure 3). We next determined whether the loss of TSC2 function in TSC2-ΔRG mice resulted in the activation of mTOR signaling in the intestine. Increased phosphorylation of S6, a marker of mTORC1 activity, is noted in TG mice compared with their WT controls as determined by immunohistochemistry (IHC) (Figure 1a). Similarly, in immunoblots from isolated intestinal mucosa, phosphorylation of S6 was increased in TG mice (Figure 1b). In agreement with the increased phosphorylation of S6, increased phosphorylation of 4E-BP, another marker of mTOR activity, was also detected in TG mice compared with WT control (Supplementary Figure 4). These results confirm that TSC2 inactivation results in the activation of mTORC1 signaling in intestinal epithelium. mTORC1 activation contributes to intestinal cell proliferation.^24, 25^ Consistently, increased cell proliferation was noted in TG mice as analyzed by Ki67 IHC staining (Supplementary Figure 5).

We showed that mTORC1 positively regulates Notch signaling in intestinal cell lines.^18^ Because Notch signaling has a critical role in the regulation of secretory cell differentiation, the effect of TSC2 inactivation on intestinal cell differentiation was next determined. MUC2 expression, a goblet cell marker, was markedly decreased in both the colon and small bowel of TSC2-mutant TG mice as noted by Alcian blue, IHC and western blotting (Figures 1c and g). Consistent with our *in vivo* findings, we previously showed that the knockdown of TSC2 decreased MUC2 expression in the intestinal cell line HT29.^19^ Together, our findings using both *in vitro* and *in vivo* models demonstrate that TSC2 is required for intestinal goblet cell differentiation.

**Figure 2:**
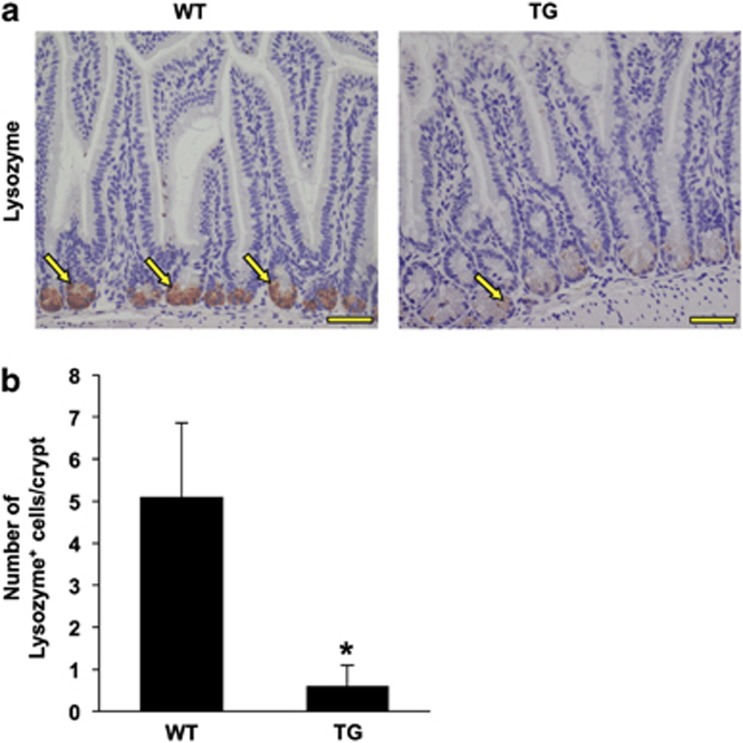
TSC2 inactivation results in altered Paneth cell differentiation in intestine. (**a**) Immunohistochemical (IHC) staining of the small intestine for lysozyme showed the decrease in Paneth cells (arrow) in TSC2-mutant TG mice compared with WT mice. (**b**) Quantification of lysozyme-positive cells in WT and TSC2-mutant TG mice. (Data represent mean±S.D.; *P<0.05 *versus* WT). Scale bars, 50 *μ*m

Remarkably, staining of intestinal sections from TSC2-mutant TG mice for lysozyme, an early marker of Paneth cell differentiation, revealed that almost all the crypts were completely devoid of Paneth cells (Figures 2a and b). The effects of TSC2 inactivation on Paneth cells have also been determined by IHC staining for MMP7, another Paneth cell marker.^26^

Consistent with the decreased lysozyme staining, decreased staining of MMP7 was also found in TSC2-mutant mice (Supplementary Figure 6). In addition, decreased enteroendocrine cell numbers were found in TSC2-mutant TG mice as assessed by chromogranin A staining (Supplementary Figure 7). Therefore, these results demonstrate that TSC2 is required for differentiation of intestinal secretory cell lineage.

### TSC2 controls intestinal cell differentiation through regulation of Notch/Hes1 signaling

**Figure 3:**
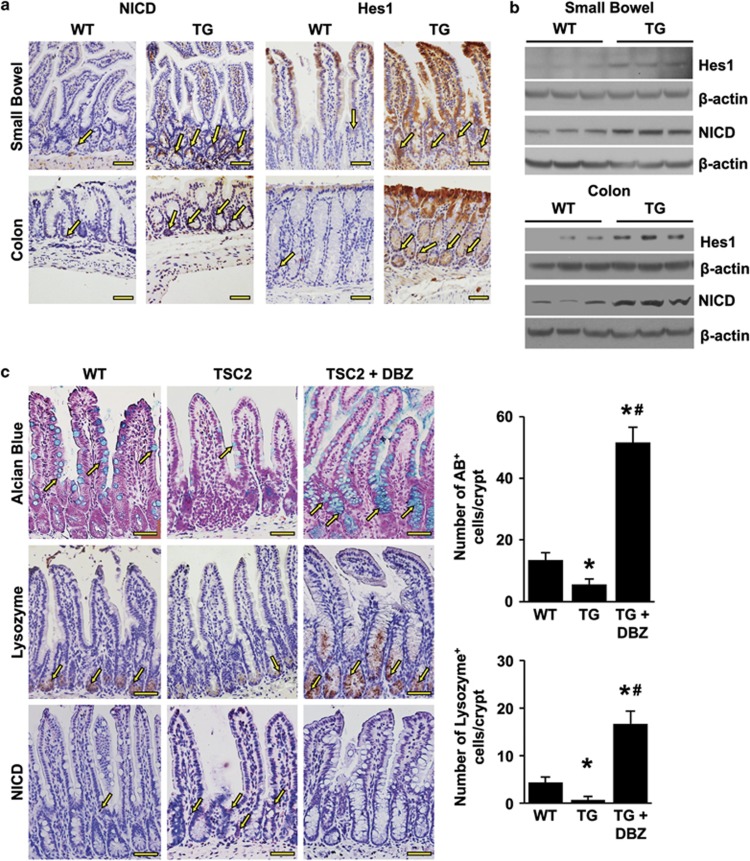
TSC2 inactivation leads to the increased Notch signaling in intestine. (**a**) Immunohistochemical (IHC) staining (arrow) for NICD and Hes1 demonstrated increased expression in the intestinal epithelium of TG mice compared with WT mice. (**b**) Mucosal protein lysates extracted from WT and TG mice were used for western blot detection of NICD and Hes1 protein expression. (**c**) TSC2-mutant TG mice were treated with or without DBZ for 5 day. The inhibition of Notch signaling by DBZ was demonstrated by staining with NICD (arrow). The goblet cell population, revealed by Alcian blue staining (arrow) and Paneth cells, assessed by IHC staining of lysozyme (arrow), are reduced in the small intestinal epithelium of TSC2-mutant TG mice. Treatment with DBZ restored the goblet and Paneth cell numbers. (Data represent mean±S.D.; *P<0.05 *versus* WT alone; ^#^*P*<0.05 *versus* TG alone). Scale bars, 50 *μ*m

Inhibition of the Notch pathway leads to an increase in intestinal secretory cell differentiation.^1, 27, 28^ Loss of TSC promotes Notch activation in *Drosophila*, rodents and humans,^29^ although proof is lacking that inactivation of TSC2 mediates Notch1 activation in intestinal cells. To determine whether TSC2 inactivation promotes Notch signaling in intestinal epithelium, we compared the expression of NICD and Hes1 in TSC2-mutant TG *versus* WT mice. The expression of NICD and Hes1 was markedly increased in the TSC2-mutant mice as shown by IHC (Figure 3a) and western blot (Figure 3b). These results demonstrate the negative regulation of Notch signaling by TSC2 in intestinal epithelium, reinforcing the notion that TSC2 controls intestinal cell differentiation through the regulation of Notch signaling.

To further demonstrate that TSC2 regulates intestinal cell differentiation through Notch signaling, we next determined whether inhibition of Notch with DBZ attenuates the repression of intestinal cell differentiation induced by TSC2 inactivation in the intestine. Administration of DBZ for 5 days effectively rescued the effect of TSC2 inactivation as shown by the increased number of goblet and Paneth cells (Figure 3c). The inhibition of Notch signaling was confirmed by the decreased NICD expression. These data demonstrate that TSC2 regulates goblet and Paneth cell differentiation through Notch signaling.

### TSC2 regulates intestinal cell differentiation via inhibition of mTOR signaling

**Figure 4:**
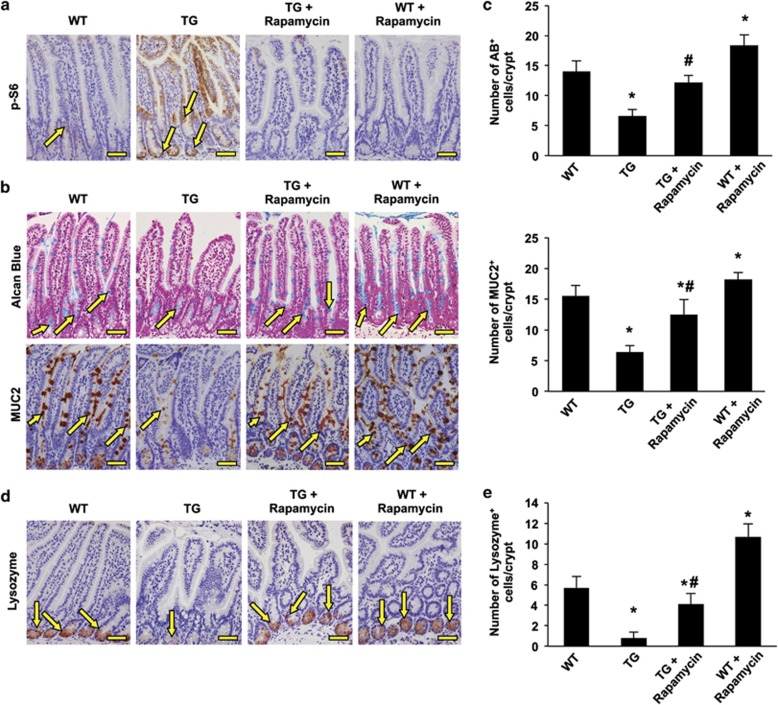
TSC2/mTOR signaling pathway controls differentiation of the goblet and Paneth cell lineages. WT- and TSC2-mutant TG mice were treated with or without rapamycin for 6 days. The inhibition of mTORC1 signaling by rapamycin was demonstrated by staining with phospho-S6 (**a**, arrow). The goblet cell population, revealed by Alcian blue staining and MUC2 immunostaining (**b**, arrow; **c**), and Paneth cells, assessed by immunohistochemical staining of lysozyme (**d**, arrow; **e**), are reduced in the small intestinal epithelium of TSC2-mutant TG mice. (Data represent mean±S.D.; *P<0.05 *versus* WT alone; #*P*<0.05 *versus* TG alone). Treatment with rapamycin restored the goblet and Paneth cell numbers. Scale bars, 50 *μ*m

Because we previously showed that REDD1/TSC2/mTOR signaling pathway regulates the expression of MUC2 in intestinal cell lines,^18, 9^ we next determined whether the inhibition of mTOR with rapamycin attenuates the repression of goblet and Paneth cell differentiation induced by TSC2 inactivation in the intestine. Administration of rapamycin for 6 days decreased phosphorylated S6 expression and, importantly, increased goblet cells in the intestine, thus effectively rescuing the effect of TSC2 inactivation (Figures 4a and c). Similarly, Paneth cells were also increased by administration of rapamycin (Figures 4d and e). These data indicate that TSC2 regulates goblet and Paneth cell differentiation through mTOR signaling.

### TSC2/mTOR signaling regulates intestinal cell differentiation by interfering with Notch signaling in intestinal epithelium

**Figure 5:**
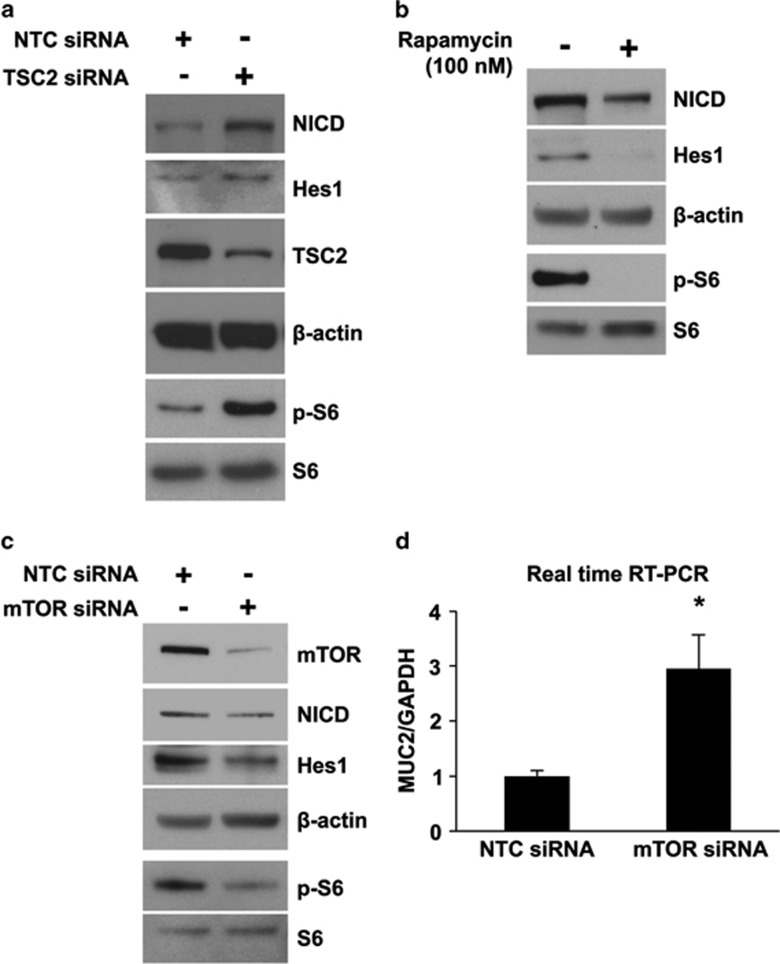
TSC2/mTOR is an upstream regulator of Notch-Hes1 signaling in the human colon cancer cell line LS174T. (**a**) LS174T cells were transfected with non-targeting control (NTC) siRNA or siRNA-targeting TSC2. (**b**) LS174T cells were treated with 100 nM rapamycin for 24 h. (**c**) LS174T cells were transfected with NTC siRNA or siRNA-targeting mTOR. Total protein was extracted and western blotting performed using anti-NICD, anti-Hes1, anti-TSC2, anti-mTOR, anti-p-S6, anti-S6 and anti-*β*-actin antibodies. (**d**) LS174T cells were transfected with NTC siRNA or siRNA-targeting mTOR; total RNA was extracted and MUC2 mRNA levels were determined by real-time RT-PCR. (Data represent mean±S.D.; *P<0.05 *versus* NTC siRNA)

TSC2 negatively regulates Notch1 signaling in an mTOR-dependent and -independent fashion.^30, 31^ To address whether TSC2 inactivation leads to the increase of Notch signaling through mTOR activation, we first transfected the human colon cancer cell line LS174 with siRNA-targeting TSC2 or non-targeting control (NTC) siRNA (Figure 5a). Knockdown of TSC2 increased the Notch transactivator NICD domain and Hes1 expression, suggesting TSC2-negative regulation of Notch signaling in LS174T cells. We next treated HT29 cells with rapamycin and, as shown in Figure 5b, treatment with rapamycin decreased the Notch transactivator NCID domain and Hes1 expression, suggesting the regulation of Notch signaling by mTOR. To further confirm the regulation of Notch by mTOR, LS174T cells were transfected with siRNA-targeting mTOR and the expression of NICD and Hes1 was determined. Knockdown of mTOR decreased the protein expression of NICD and Hes1, demonstrating mTOR-positive regulation of Notch signaling (Figure 5c). To determine whether mTOR regulates the expression of MUC2, RNA from LS174T cells transfected with either NTC siRNA or mTOR siRNA was used for analysis of MUC2 mRNA expression by real-time RT-PCR. Knockdown of mTOR significantly increased MUC2 mRNA expression in LS174T cells (Figure 5d). These results demonstrate the regulation of MUC2 expression by the TSC2/mTOR/Notch signaling pathway in LS174T cells.

**Figure 6:**
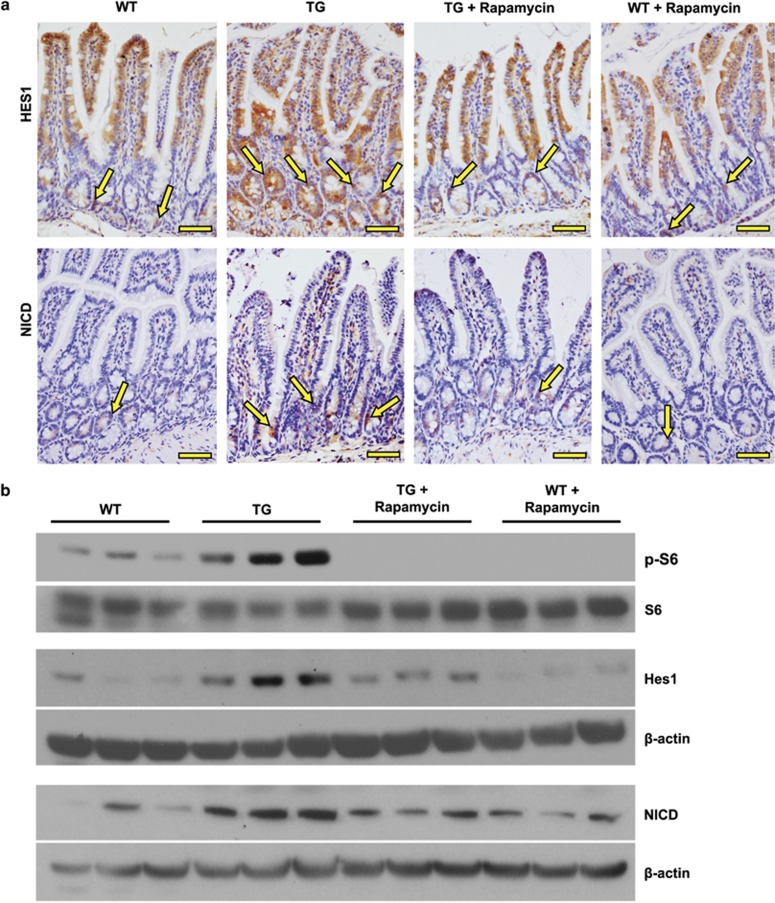
TSC2/mTOR signaling pathway regulates intestinal cell differentiation through Notch1. (**a**) WT and TG mice were treated with or without rapamycin for 6 days. The decreased immunohistochemical staining for NICD and Hes1 (arrow) demonstrated the inhibition of Notch signaling by rapamycin in the intestinal epithelium. (**b**) Mucosal protein lysates extracted from WT and TG mice treated with or without rapamycin for 6 days were used for western blot detection of NICD and Hes1 protein expression. Scale bars, 50 *μ*m

Finally, we determined whether inhibition of mTOR with rapamycin attenuated the increased NICD and Hes1 expression mediated by TSC2 inactivation in mouse intestinal epithelium. As shown in Figure 6, TSC2 inactivation leads to increased NICD and Hes1 expression as expected; this increase was significantly attenuated by administration of rapamycin as shown by IHC (Figure 6a) and western blot (Figure 6b). Taken together, our results demonstrate the regulation of intestinal goblet and Paneth cell differentiation by the TSC2/mTOR/Notch signaling pathway.

## Discussion

**Figure 7:**
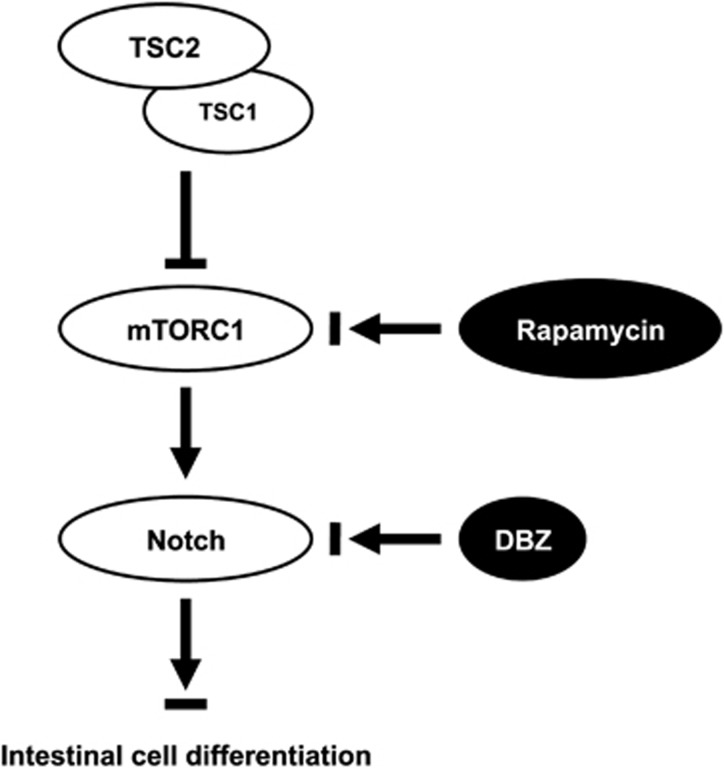
Schematic representation of TSC2/mTOR/Notch pathway model. TSC2 functions as a complex with TSC1 and inhibits mTORC1 signaling, thus leading to decreased Notch signaling and increased intestinal cell differentiation

The upstream mechanisms that initiate and control intestinal differentiation remain largely undefined. We showed that activation of mTORC1 decreased, whereas inhibition of mTORC1 increased, goblet cell differentiation in human intestinal cell lines.^18, 19^ We now provide evidence and demonstrate the role of TSC2/mTOR in the differentiation of intestinal cells *in vivo*. Mutant TSC2 mice demonstrate an activation of mTORC1 and Notch signaling along with an obvious decrease in goblet and Paneth cells in the intestine; this reduction was markedly attenuated by DBZ or rapamycin treatment. Consistently, knockdown of TSC2 activates, whereas knockdown of mTOR inhibits, Notch signaling in the human colon cancer cell line LS174T. Taken together, our results demonstrate that intestinal cell differentiation is regulated by TSC2/mTOR/Notch signaling (Figure 7).

mTOR has been implicated in the signaling pathways regulating cell growth, apoptosis and differentiation of various cell types.^32, 33, 34^ Recently, we showed that inhibition of mTORC1 contributes to goblet cell differentiation in human intestinal cell lines.^18, 19^ In our present study, we show that the TSC2/mTORC1 signaling pathway has a critical role in the regulation of intestinal goblet and Paneth cell differentiation in mice. Consistent with our findings, Yilmaz *et al.*,^35^ showed that administration of rapamycin for 4 weeks increased the frequency of Paneth cells in the mouse intestine. Moreover, loss of A*denomatous polyposis coli* (APC) in mice results in an absence of goblet cells; conversely, treatment with rapamycin restored goblet cell numbers.^25^ HDAC1- and HDAC2-deficient mice demonstrated increased mTORC1 activity correlating with reduction of goblet and Paneth cells in the intestine.^36^ Furthermore, dysregulated differentiation within the intestinal crypts is associated with the progression of colorectal cancer.^5^ We previously demonstrated elevated mTOR activity and overexpression of mTOR complex components in CRC.^37, 38^ Treatment with rapamycin has been shown to inhibit the polyposis and progression to dysplasia induced by APC mutation.^25^ Taken together, our studies and those of others^25, 35, 36^ demonstrate the regulation of intestinal differentiation by mTORC1 signaling.

The interaction between TSC2/mTOR and Notch signaling has been noted in various cell types. On the one hand, TSC2/mTOR acts as an upstream regulator of Notch signaling. Our results show that TSC2 inhibits mTORC1, leading to the inhibition of Notch signaling in intestinal cells. Similarly, TSC2 has been shown to inhibit Notch through the inhibition of mTORC1 signaling in MEF cells and in some cancer cell lines.^30^ In addition, TSC2 has been shown to inhibit Notch in an mTOR-independent fashion.^31^ Conversely, Notch signaling increases mTOR signaling in certain cell types. For example, Notch signaling promotes hepatic lipogenesis through activation of mTORC1.^39^ In addition, Notch promotes mTORC1 signaling by increasing the levels of Raptor, a core component of mTORC1, as well as mTORC1 assembly. Notch also suppresses TSC2 expression and regulates cell differentiation in the *Drosophila* intestinal stem cell lineage.^40^ Together, these studies suggest that two major biochemical pathways, the TSC2/mTOR pathway and the Notch/Hes1 pathway, cooperate to control cellular differentiation *in vivo*. It remains to be seen whether Notch signaling also regulates TSC2/mTOR signaling in intestinal cells.

We showed that inactivation of TSC2 leads to a profound decrease of goblet and Paneth cell lineages in the intestine and that this reduction was significantly attenuated by inhibition of mTORC1 using rapamycin. However, these decreased goblet and Paneth cells were not completely restored by mTOR inhibition. As TSC2 has been shown to inhibit Notch in an mTOR-independent fashion,^31^ inactivation of TSC2 may also activate Notch signaling independent of mTOR regulation in intestinal cells. Indeed, deletion of LKB1 results in the activation of Notch-Hes5 signaling and thus alteration of intestinal goblet and Paneth cell differentiation through an mTOR-independent fashion.^41^ The LKB1/AMPK signaling cascade negatively regulates mTOR by phosphorylating and activating TSC2.^42^ Therefore, TSC2 likely inhibits Notch and regulates intestinal goblet and Paneth cell differentiation through mechanisms that are both dependent and independent of mTOR.

IBD, most notably Crohn's disease (CD) and ulcerative colitis (UC), is associated with defects in stem cell differentiation, resulting in a decrease of Paneth cells in CD or goblet cells in UC.^4, 43^ Paneth cells utilize the secretion of defensins and other microbicidal peptides to kill invasive pathogens, shape the intestinal microbiota and protect intestinal stem cells from harm.^44^ Mucins, secreted by goblet cells, protect the epithelial surface of the GI tract by forming a semi-permeable mucous layer between the lumen and the intestinal epithelium.^45, 46^ Diminished Paneth cell differentiation results in a defective antimicrobial barrier in CD, whereas in UC, an insufficient induction of goblet cell mucins can lead to a disturbed mucosal barrier. Elevated mTOR activity has been identified in the colonic epithelial cells of human IBD patients with active disease.^47^ Inhibitors of TORC1 have proven to be effective in IBD.^48, 49^ In addition, there are two case reports, indicating that the mTOR inhibitors, sirolimus and everolimus, induce remission in refractory CD patients.^50, 51^ These findings suggest that targeting mTOR may offer a promising therapeutic strategy for the treatment of IBD.

In conclusion, we report that TSC2/mTOR signaling regulates intestinal cell differentiation in a Notch-dependent manner, thus providing a better mechanistic understanding of mTOR inhibition and its potential beneficial effects in certain intestinal diseases. Our current *in vivo* findings further expand our earlier *in vitro* reports in which knockdown of TSC2 inhibited, whereas inhibition of mTOR increased, goblet cell differentiation in colon cancer cells. Taken together, our data support a novel role of TSC2/mTOR in the regulation of Notch signaling and the maintenance of intestinal epithelial homeostasis.

## Materials and Methods

### Mice

C57BL/6 and TSC2- ΔRG TG mice (strain name C57BL/6-Tg (CMV-Tsc2*) 1 Arbi/KlanJ, stock number 014564) were purchased from the Jackson Laboratory (Sacramento, CA, USA), maintained on a 12-h light/dark schedule in filter top isolators with autoclaved water under specific pathogen-free conditions, and fed autoclaved standard laboratory chow *ad libitum*. Genotypes of mice were determined from DNA isolated by ear punch with the use of DNeasy Blood & Tissue Kit (Qiagen, Valencia, CA, USA). DBZ (Selleckchem, Radnor, PA, USA), was injected intraperitoneally (i.p.) daily for 5 days at 30 *μ*mol/kg. DBZ was finely suspended in 0.5% (w/v) hydroxypropylmethylcellulose (Methocel E4M) and 0.1% (w/v) Tween 80 in water as described.^28^ Rapamycin (LC Laboratories, Woburn, MA, USA), administered by i.p. injection daily for 6 days at 4 mg/kg, was reconstituted in absolute ethanol at 10 mg/ml and diluted in 5% Tween 80 (Sigma) and 5% PEG-400 (Hampton Research, Aliso Viejo, CA, USA) before injection as described previously.^35^ The final volume of all injections was 200 *μ*l. The ileum and cecum from TSC2-mutant and WT mice were harvested, opened and washed with ice-cold PBS. Half of the sample was used for IHC; the mucosa from the other portion was scraped with glass slides, placed into cell lysis buffer and immediately snap frozen in liquid N_2_. Samples were homogenized in cell lysis buffer by stainless steel blend bead beating (0.9–2.0 mm; 5 m, 4 °C) using a Bullet Blender (Next Advance Inc, Averill Park, NY, USA). Cell lysis buffer (Cell Signaling, Beverly, MA, USA) was supplemented with 1 mM PMSF and protease inhibitor cocktails (complete mini and complete ultra, EDTA-free; one tablet per 10 ml lysis buffer; Roche, Indianapolis, IN, USA). All animal procedures were conducted with approval and in compliance with University of Kentucky Institutional Animal Care and Use Committee.

### Cell culture, transfection and treatment

The human colon cancer cell line, LS174T, was maintained in RPMI supplemented with 5% FCS. Cells were transfected with the siRNA duplexes by electroporation (Gene Pulser, Bio-Rad, Hercules, CA, USA) as we have described previously.^52, 53^ Human TSC2, mTOR and NTC siRNA SMARTpool were purchased from Dharmacon, Inc. (Lafayette, CO, USA). siRNA SMARTpool, consisting of four siRNA duplexes, was designed using an algorithm comprised of 33 criteria and parameters that effectively eliminate non-functional siRNA.^54^

### Western blot analysis

Total protein was resolved on a 10% polyacrylamide gel and transferred to polyvinylidene fluoride membranes. Membranes were incubated for 1 h at room temperature in blotting solution. Antibodies to TSC2 (Santa Cruz, CA, USA), mTOR, phospho-S6 (pS235/236), S6 and phospho-4E-BP1 (Thr37/46) (all from Cell Signaling), MUC2 and Notch1 (NICD) (both from Epitomics Inc., Burlingame, CA, USA), Hes1 (Millipore, Billerica, MA, USA) and *β*-actin (Sigma, St. Louis, MO, USA) were added, and following blotting with a horseradish peroxidase-conjugated secondary antibody, protein expression was visualized using an enhanced chemiluminescence detection system.

### Quantitative real-time RT-PCR analysis

Total RNA was extracted and treated with DNase (RQ1, Promega, Madison, WI, USA). Synthesis of cDNA was performed with 1 *μ*g of total RNA using reagents in the TaqMan Reverse Transcription Reagents Kit (ABI #N8080234). TaqMan probe and primers for human MUC2 and GAPDH were purchased from Applied Biosystems (Foster City, CA, USA). Quantitative real-time RT-PCR analysis was performed with an Applied Biosystems Prism 7000HT Sequence Detection System using TaqMan universal PCR master mix as we have described previously.^55^

### IHC and Alcian Blue (AB) staining and TUNEL assay

Tissue was processed for routine IHC staining using the following antibodies: rabbit anti-Lysozyme (Diagnostic BioSystems, Pleasanton, CA, USA; RP 028-05), rabbit monoclonal anti-Notch1 (NICD) antibody (Epitomics Inc. 1935-1), anti-Hes1 (Millipore, 5702), anti-MUC2 (Santa Cruz, SC15334), anti-phospho-S6 (pS235/236) (Cell Signaling, #4858), rabbit chromogranin A (Abcam, Cambridge, MA, USA; 15160), mouse MMP7 (R&D Systems, Minneapolis, MN, USA; AF2967) and rabbit Ki67 (Novus, Littleton, CO, USA; NB110-89717). Negative controls (including no primary antibody or isotype-matched mouse immunoglobulin G) were used in each assessment. AB staining was performed according to standard protocol using AB pH 2.5 Stain Kit (Dako, Carpinteria, CA, USA; AR160). Apoptotic cells were detected by TUNEL assay using an ApopTag Peroxidase *In Situ* Apoptosis Detection Kit (Millipore, S7100).

### Statistical analysis

Comparisons of the number of AB+, MUC2+, lysozyme+ in the intestine were performed between WT- and TSC2-mutant mice using the linear mixed model to account for multiple observations from multiple crypts per mouse. Pairwise comparisons, specified *a priori*, were performed using contrast statements from the model. Two-sample *t*-test was used for comparison of qRT-PCR between control siRNA *versus* mTOR siRNA. Bar graphs represent mean±S.D. levels in each group. *P*-values <0.05 were considered statistically significant.

## Abbreviations

TSC: tuberous sclerosis complex
NICD: notch intracellular domain
Hes1: hairy/enhancer of split 1
RT-PCR: reverse transcription polymerase chain reaction
siRNA: small interfering RNA

## References

[bib1] 1Yeung TM, Chia LA, Kosinski CM, Kuo CJ. Regulation of self-renewal and differentiation by the intestinal stem cell niche. Cell Mol Life Sci 2011; 68: 2513–2523. 21509540 10.1007/s00018-011-0687-5 PMC4165857

[bib2] 2De Mey JR, Freund JN. Understanding epithelial homeostasis in the intestine: an old battlefield of ideas, recent breakthroughs and remaining controversies. Tissue Barriers 2013; 1: e24965. 24665395 10.4161/tisb.24965 PMC3879175

[bib3] 3Gunther C, Neumann H, Neurath MF, Becker C. Apoptosis, necrosis and necroptosis: cell death regulation in the intestinal epithelium. Gut 2013; 62: 1062–1071. 22689519 10.1136/gutjnl-2011-301364

[bib4] 4Gersemann M, Wehkamp J, Stange EF. Innate immune dysfunction in inflammatory bowel disease. J Intern Med 2012; 271: 421–428. 22324936 10.1111/j.1365-2796.2012.02515.x

[bib5] 5Hammoud SS, Cairns BR, Jones DA. Epigenetic regulation of colon cancer and intestinal stem cells. Curr Opin Cell Biol 2013; 25: 177–183. 23402869 10.1016/j.ceb.2013.01.007 PMC3615052

[bib6] 6Clark JA, Doelle SM, Halpern MD, Saunders TA, Holubec H, Dvorak K et al. Intestinal barrier failure during experimental necrotizing enterocolitis: protective effect of EGF treatment. Am J Physiol Gastrointest Liver Physiol 2006; 291: G938–G949. 16798726 10.1152/ajpgi.00090.2006

[bib7] 7Curatolo P, Bombardieri R, Jozwiak S. Tuberous sclerosis. Lancet 2008; 372: 657–668. 18722871 10.1016/S0140-6736(08)61279-9

[bib8] 8Tee AR, Fingar DC, Manning BD, Kwiatkowski DJ, Cantley LC, Blenis J. Tuberous sclerosis complex-1 and -2 gene products function together to inhibit mammalian target of rapamycin (mTOR)-mediated downstream signaling. Proc Natl Acad Sci USA 2002; 99: 13571–13576. 12271141 10.1073/pnas.202476899 PMC129715

[bib9] 9Gingras AC, Raught B, Sonenberg N. Regulation of translation initiation by FRAP/mTOR. Genes Dev 2001; 15: 807–826. 11297505 10.1101/gad.887201

[bib10] 10Makky K, Tekiela J, Mayer AN. Target of rapamycin (TOR) signaling controls epithelial morphogenesis in the vertebrate intestine. Dev Biol 2007; 303: 501–513. 17222402 10.1016/j.ydbio.2006.11.030 PMC2715143

[bib11] 11DeYoung MP, Horak P, Sofer A, Sgroi D, Ellisen LW. Hypoxia regulates TSC1/2-mTOR signaling and tumor suppression through REDD1-mediated 14-3-3 shuttling. Genes Dev 2008; 22: 239–251. 18198340 10.1101/gad.1617608 PMC2192757

[bib12] 12Guertin DA, Sabatini DM. Defining the role of mTOR in cancer. Cancer Cell 2007; 12: 9–22. 17613433 10.1016/j.ccr.2007.05.008

[bib13] 13Bray SJ. Notch signalling: a simple pathway becomes complex. Nat Rev Mol Cell Biol 2006; 7: 678–689. 16921404 10.1038/nrm2009

[bib14] 14Stanger BZ, Datar R, Murtaugh LC, Melton DA. Direct regulation of intestinal fate by Notch. Proc Natl Acad Sci USA 2005; 102: 12443–12448. 16107537 10.1073/pnas.0505690102 PMC1194941

[bib15] 15Jensen J, Pedersen EE, Galante P, Hald J, Heller RS, Ishibashi M et al. Control of endodermal endocrine development by Hes-1. Nat Genet 2000; 24: 36–44. 10615124 10.1038/71657

[bib16] 16Ueo T, Imayoshi I, Kobayashi T, Ohtsuka T, Seno H, Nakase H et al. The role of Hes genes in intestinal development, homeostasis and tumor formation. Development 2012; 139: 1071–1082. 22318232 10.1242/dev.069070

[bib17] 17Rodilla V, Villanueva A, Obrador-Hevia A, Robert-Moreno A, Fernandez-Majada V, Grilli A et al. Jagged1 is the pathological link between Wnt and Notch pathways in colorectal cancer. Proc Natl Acad Sci USA 2009; 106: 6315–6320. 19325125 10.1073/pnas.0813221106 PMC2669348

[bib18] 18Zhou Y, Wang Q, Weiss HL, Evers BM. Nuclear factor of activated T-cells 5 increases intestinal goblet cell differentiation through an mTOR/Notch signaling pathway. Mol Biol Cell 2014; 25: 2882–2890. 25057011 10.1091/mbc.E14-05-0998 PMC4161521

[bib19] 19Zhou Y, Wang Q, Guo Z, Weiss HL, Evers BM. Nuclear factor of activated T-cell c3 inhibition of mammalian target of rapamycin signaling through induction of regulated in development and DNA damage response 1 in human intestinal cells. Mol Biol Cell 2012; 23: 2963–2972. 22696685 10.1091/mbc.E12-01-0037 PMC3408422

[bib20] 20Onda H, Lueck A, Marks PW, Warren HB, Kwiatkowski DJ. Tsc2(+/-) mice develop tumors in multiple sites that express gelsolin and are influenced by genetic background. J Clin Invest 1999; 104: 687–695. 10491404 10.1172/JCI7319 PMC408440

[bib21] 21Bhatia B, Northcott PA, Hambardzumyan D, Govindarajan B, Brat DJ, Arbiser JL et al. Tuberous sclerosis complex suppression in cerebellar development and medulloblastoma: separate regulation of mammalian target of rapamycin activity and p27 Kip1 localization. Cancer Res 2009; 69: 7224–7234. 19738049 10.1158/0008-5472.CAN-09-1299 PMC2745891

[bib22] 22Govindarajan B, Brat DJ, Csete M, Martin WD, Murad E, Litani K et al. Transgenic expression of dominant negative tuberin through a strong constitutive promoter results in a tissue-specific tuberous sclerosis phenotype in the skin and brain. J Biol Chem 2005; 280: 5870–5874. 15576369 10.1074/jbc.M411768200

[bib23] 23Chevere-Torres I, Kaphzan H, Bhattacharya A, Kang A, Maki JM, Gambello MJ et al. Metabotropic glutamate receptor-dependent long-term depression is impaired due to elevated ERK signaling in the DeltaRG mouse model of tuberous sclerosis complex. Neurobiol Dis 2012; 45: 1101–1110. 22198573 10.1016/j.nbd.2011.12.028 PMC3276695

[bib24] 24Shao J, Evers BM, Sheng H. Roles of phosphatidylinositol 3'-kinase and mammalian target of rapamycin/p70 ribosomal protein S6 kinase in K-Ras-mediated transformation of intestinal epithelial cells. Cancer Res 2004; 64: 229–235. 14729629 10.1158/0008-5472.can-03-1859

[bib25] 25Hardiman KM, Liu J, Feng Y, Greenson JK, Fearon ER. Rapamycin inhibition of polyposis and progression to dysplasia in a mouse model. PLoS One 2014; 9: e96023. 24763434 10.1371/journal.pone.0096023 PMC3999114

[bib26] 26Schneider MR, Dahlhoff M, Horst D, Hirschi B, Trulzsch K, Muller-Hocker J et al. A key role for E-cadherin in intestinal homeostasis and Paneth cell maturation. PLoS One 2010; 5: e14325. 21179475 10.1371/journal.pone.0014325 PMC3001873

[bib27] 27Vooijs M, Liu Z, Kopan R. Notch: architect, landscaper, and guardian of the intestine. Gastroenterology 2011; 141: 448–459. 21689653 10.1053/j.gastro.2011.06.003 PMC4050496

[bib28] 28VanDussen KL, Carulli AJ, Keeley TM, Patel SR, Puthoff BJ, Magness ST et al. Notch signaling modulates proliferation and differentiation of intestinal crypt base columnar stem cells. Development 2012; 139: 488–497. 22190634 10.1242/dev.070763 PMC3252352

[bib29] 29Pear WS. New roles for Notch in tuberous sclerosis. J Clin Invest 2010; 120: 84–87. 20038806 10.1172/JCI41897 PMC2798710

[bib30] 30Ma J, Meng Y, Kwiatkowski DJ, Chen X, Peng H, Sun Q et al. Mammalian target of rapamycin regulates murine and human cell differentiation through STAT3/p63/Jagged/Notch cascade. J Clin Invest 2010; 120: 103–114. 20038814 10.1172/JCI37964 PMC2798675

[bib31] 31Karbowniczek M, Zitserman D, Khabibullin D, Hartman T, Yu J, Morrison T et al. The evolutionarily conserved TSC/Rheb pathway activates Notch in tuberous sclerosis complex and Drosophila external sensory organ development. J Clin Invest 2010; 120: 93–102. 20038815 10.1172/JCI40221 PMC2798691

[bib32] 32Lamming DW, Sabatini DM. A Central role for mTOR in lipid homeostasis. Cell Metab 2013; 18: 465–469. 23973332 10.1016/j.cmet.2013.08.002 PMC3818790

[bib33] 33Dai J, Bercury KK, Macklin WB. Interaction of mTOR and Erk1/2 signaling to regulate oligodendrocyte differentiation. Glia 2014; 62: 2096–2109. 25060812 10.1002/glia.22729 PMC4406223

[bib34] 34Laplante M, Sabatini DM. mTOR signaling in growth control and disease. Cell 2012; 149: 274–293. 22500797 10.1016/j.cell.2012.03.017 PMC3331679

[bib35] 35Yilmaz OH, Katajisto P, Lamming DW, Gultekin Y, Bauer-Rowe KE, Sengupta S et al. mTORC1 in the Paneth cell niche couples intestinal stem-cell function to calorie intake. Nature 2012; 486: 490–495. 22722868 10.1038/nature11163 PMC3387287

[bib36] 36Turgeon N, Blais M, Gagne JM, Tardif V, Boudreau F, Perreault N et al. HDAC1 and HDAC2 restrain the intestinal inflammatory response by regulating intestinal epithelial cell differentiation. PLoS One 2013; 8: e73785. 24040068 10.1371/journal.pone.0073785 PMC3764035

[bib37] 37Johnson SM, Gulhati P, Rampy BA, Han Y, Rychahou PG, Doan HQ et al. Novel expression patterns of PI3K/Akt/mTOR signaling pathway components in colorectal cancer. J Am Coll Surg 2010; 210: 776–768. 10.1016/j.jamcollsurg.2009.12.008 PMC2895913 20421047

[bib38] 38Gulhati P, Cai Q, Li J, Liu J, Rychahou PG, Qiu S et al. Targeted inhibition of mammalian target of rapamycin signaling inhibits tumorigenesis of colorectal cancer. Clin Cancer Res 2009; 15: 7207–7216. 19934294 10.1158/1078-0432.CCR-09-1249 PMC2898570

[bib39] 39Pajvani UB, Qiang L, Kangsamaksin T, Kitajewski J, Ginsberg HN, Accili D. Inhibition of Notch uncouples Akt activation from hepatic lipid accumulation by decreasing mTorc1 stability. Nat Med 2013; 19: 1054–1060. 23832089 10.1038/nm.3259 PMC3737382

[bib40] 40Kapuria S, Karpac J, Biteau B, Hwangbo D, Jasper H. Notch-mediated suppression of TSC2 expression regulates cell differentiation in the Drosophila intestinal stem cell lineage. PLoS Genet 2012; 8: e1003045. 23144631 10.1371/journal.pgen.1003045 PMC3493453

[bib41] 41Shorning BY, Zabkiewicz J, McCarthy A, Pearson HB, Winton DJ, Sansom OJ et al. Lkb1 deficiency alters goblet and paneth cell differentiation in the small intestine. PLoS One 2009; 4: e4264. 19165340 10.1371/journal.pone.0004264 PMC2626247

[bib42] 42Shaw RJ. LKB1 and AMP-activated protein kinase control of mTOR signalling and growth. Acta Physiol (Oxf) 2009; 196: 65–80. 19245654 10.1111/j.1748-1716.2009.01972.x PMC2760308

[bib43] 43Gersemann M, Stange EF, Wehkamp J. From intestinal stem cells to inflammatory bowel diseases. World J Gastroenterol 2011; 17: 3198–3203. 21912468 10.3748/wjg.v17.i27.3198 PMC3158395

[bib44] 44Salzman NH, Bevins CL. Dysbiosis—a consequence of Paneth cell dysfunction. Semin Immunol 2013; 25: 334–341. 24239045 10.1016/j.smim.2013.09.006

[bib45] 45Shirazi T, Longman RJ, Corfield AP, Probert CS. Mucins and inflammatory bowel disease. Postgrad Med J 2000; 76: 473–478. 10908374 10.1136/pmj.76.898.473 PMC1741691

[bib46] 46Einerhand AW, Renes IB, Makkink MK, van der Sluis M, Buller HA, Dekker J. Role of mucins in inflammatory bowel disease: important lessons from experimental models. Eur J Gastroenterol Hepatol 2002; 14: 757–765. 12169985 10.1097/00042737-200207000-00008

[bib47] 47Deng L, Zhou JF, Sellers RS, Li JF, Nguyen AV, Wang Y et al. A novel mouse model of inflammatory bowel disease links mammalian target of rapamycin-dependent hyperproliferation of colonic epithelium to inflammation-associated tumorigenesis. Am J Pathol 2010; 176: 952–967. 20042677 10.2353/ajpath.2010.090622 PMC2808099

[bib48] 48Yin H, Li X, Zhang B, Liu T, Yuan B, Ni Q et al. Sirolimus ameliorates inflammatory responses by switching the regulatory T/T helper type 17 profile in murine colitis. Immunology 2013; 139: 494–502. 23480027 10.1111/imm.12096 PMC3719066

[bib49] 49Garcia-Maurino S, Alcaide A, Dominguez C. Pharmacological control of autophagy: therapeutic perspectives in inflammatory bowel disease and colorectal cancer. Curr Pharm Des 2012; 18: 3853–3873. 22632751 10.2174/138161212802083653

[bib50] 50Dumortier J, Lapalus MG, Guillaud O, Poncet G, Gagnieu MC, Partensky C et al. Everolimus for refractory Crohn's disease: a case report. Inflamm Bowel Dis 2008; 14: 874–877. 18275074 10.1002/ibd.20395

[bib51] 51Massey DC, Bredin F, Parkes M. Use of sirolimus (rapamycin) to treat refractory Crohn's disease. Gut 2008; 57: 1294–1296. 18719139 10.1136/gut.2008.157297

[bib52] 52Wang Q, Wang X, Evers BM. Induction of cIAP-2 in human colon cancer cells through PKC delta/NF-kappa B. J Biol Chem 2003; 278: 51091–51099. 14527959 10.1074/jbc.M306541200

[bib53] 53Wang Q, Zhou Y, Wang X, Evers BM. Glycogen synthase kinase-3 is a negative regulator of extracellular signal-regulated kinase. Oncogene 2006; 25: 43–50. 16278684 10.1038/sj.onc.1209004 PMC1413679

[bib54] 54Reynolds A, Leake D, Boese Q, Scaringe S, Marshall WS, Khvorova A. Rational siRNA design for RNA interference. Nat Biotech 2004; 22: 326–330. 10.1038/nbt936 14758366

[bib55] 55Wang Q, Zhou Y, Rychahou P, Liu C, Weiss HL, Evers BM. NFAT5 represses canonical Wnt signaling via inhibition of beta-catenin acetylation and participates in regulating intestinal cell differentiation. Cell Death Dis 2013; 4: e671. 23764852 10.1038/cddis.2013.202 PMC3702276

