# Supplementary figures and images for: TSC2/mTORC1 signaling controls Paneth and goblet cell differentiation in the intestinal epithelium
Source: Cell Death Dis. 2015 Feb 5;6(2):e1631–. doi: 10.1038/cddis.2014.588 (PMC4669793; doi:10.1038/cddis.2014.588)

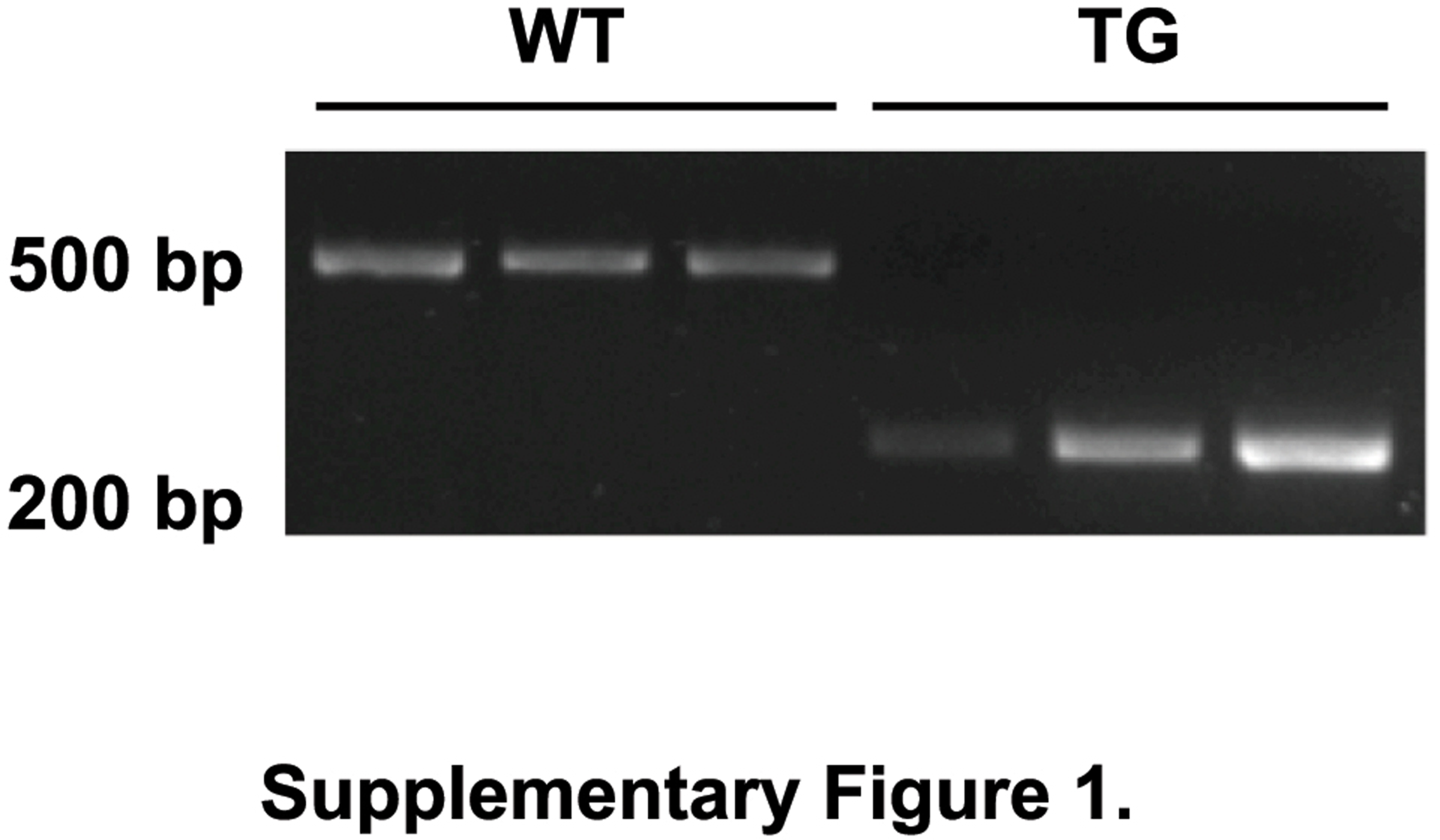

Supplement: Supplementary Figure 1 [file cddis2014588x1.tif]

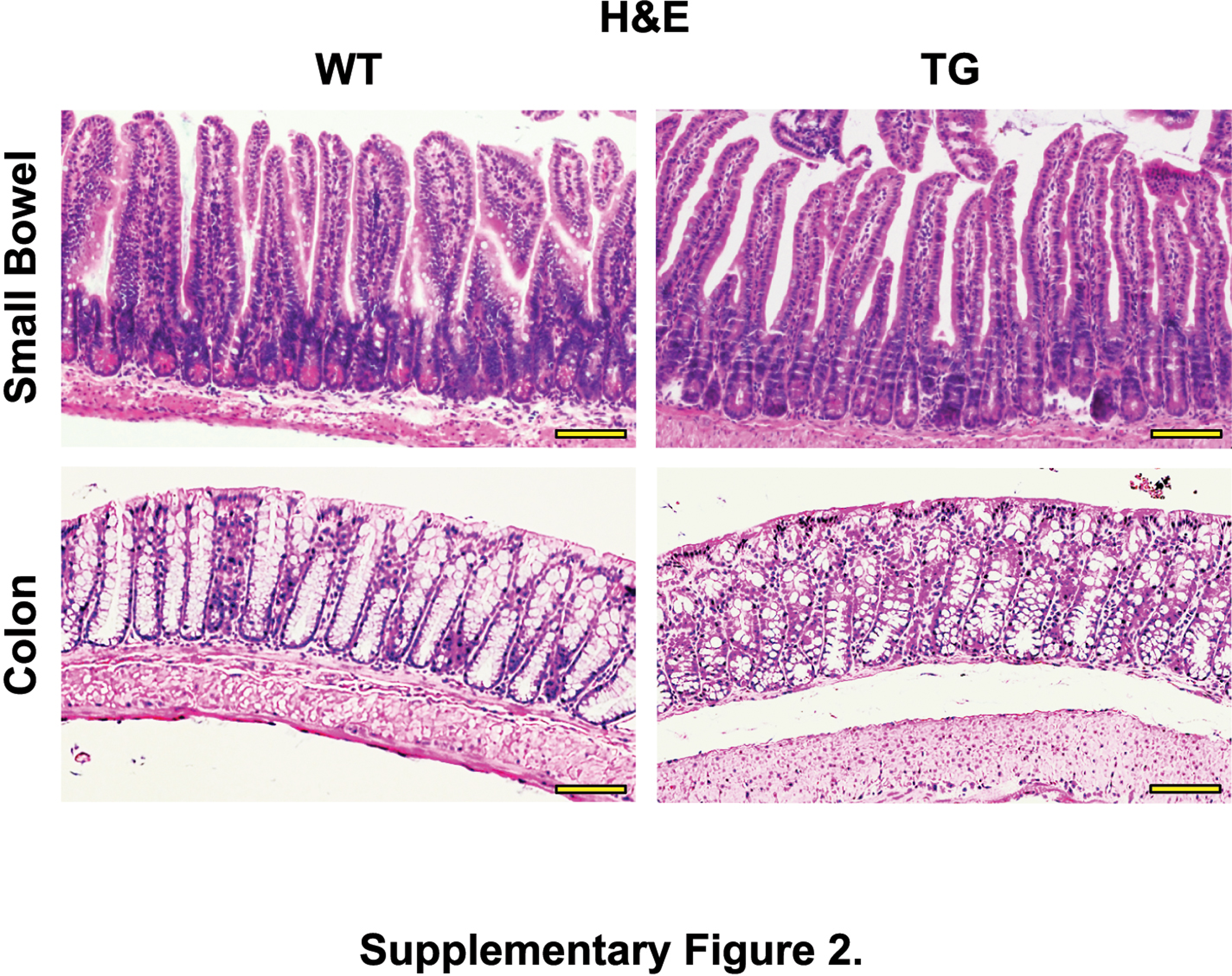

Supplement: Supplementary Figure 2 [file cddis2014588x2.tif]

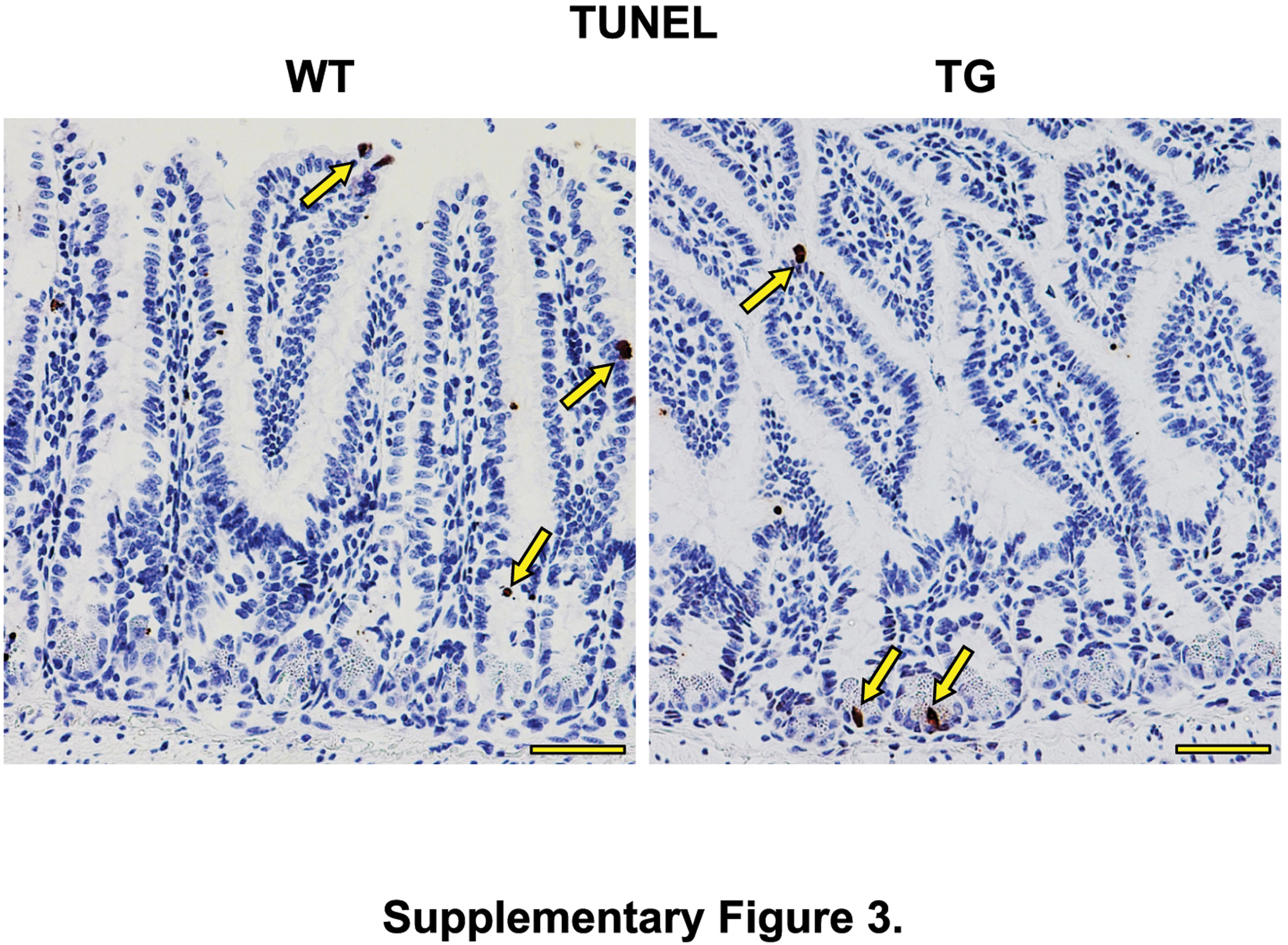

Supplement: Supplementary Figure 3 [file cddis2014588x3.tif]

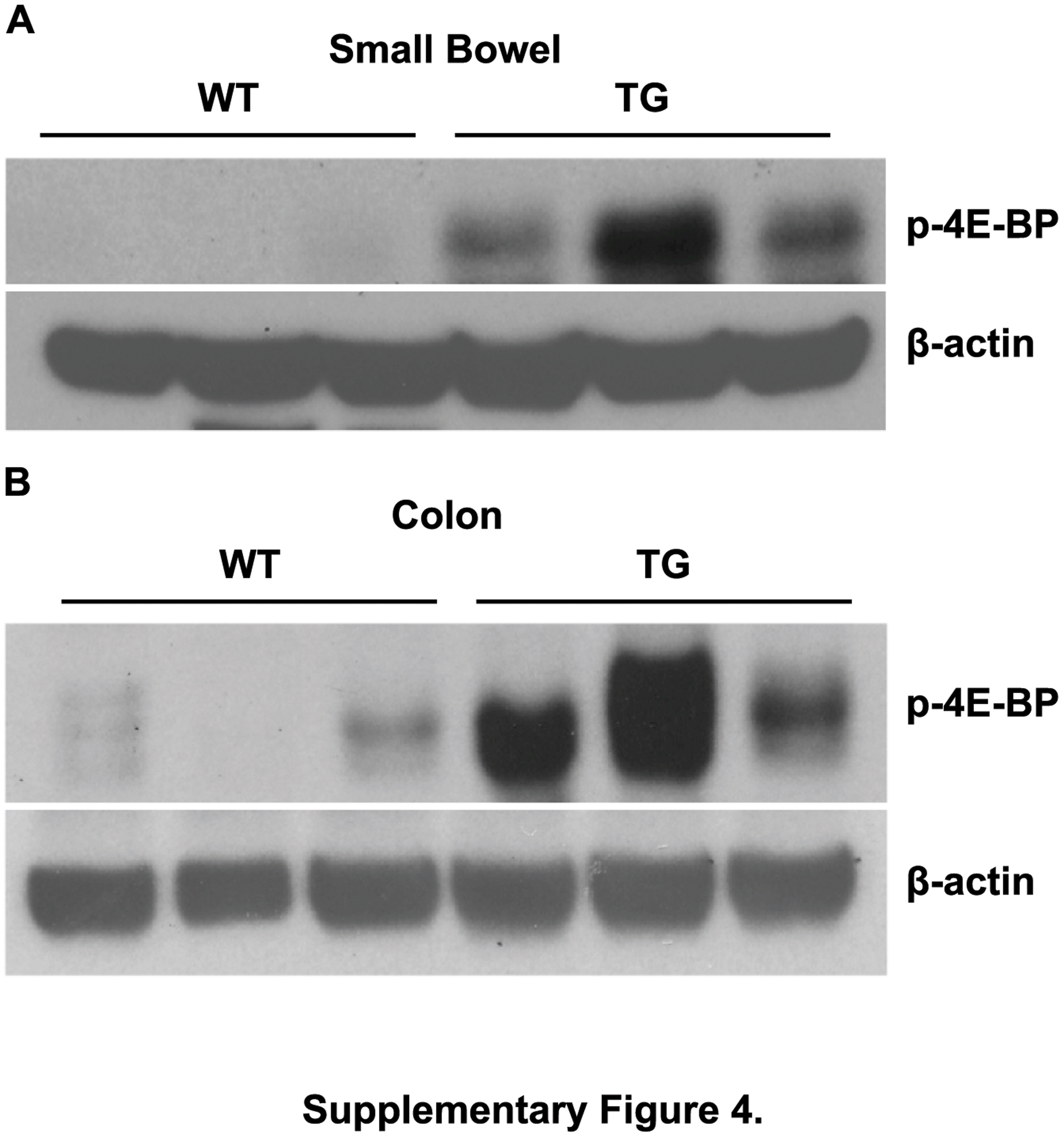

Supplement: Supplementary Figure 4 [file cddis2014588x4.tif]

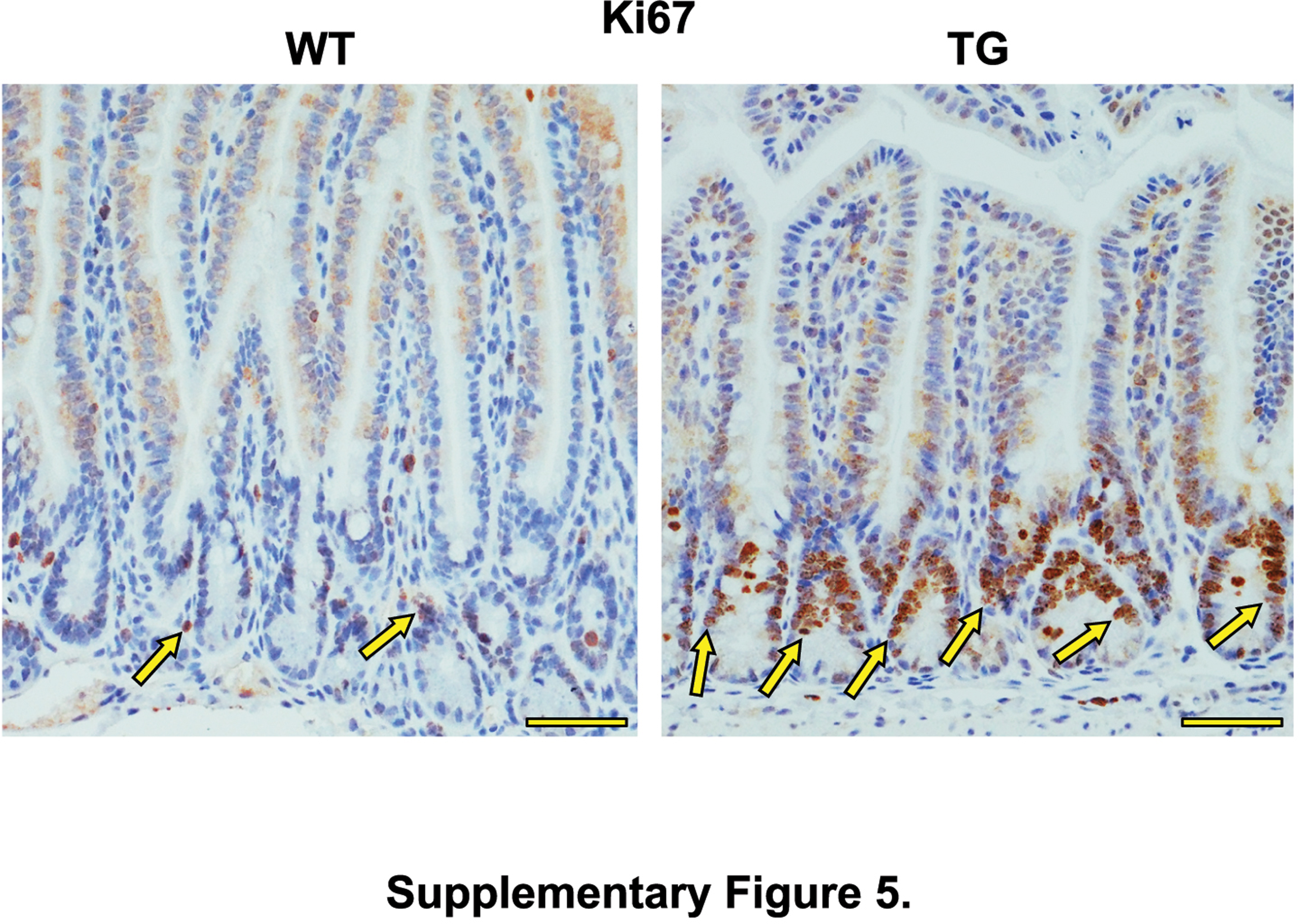

Supplement: Supplementary Figure 5 [file cddis2014588x5.tif]

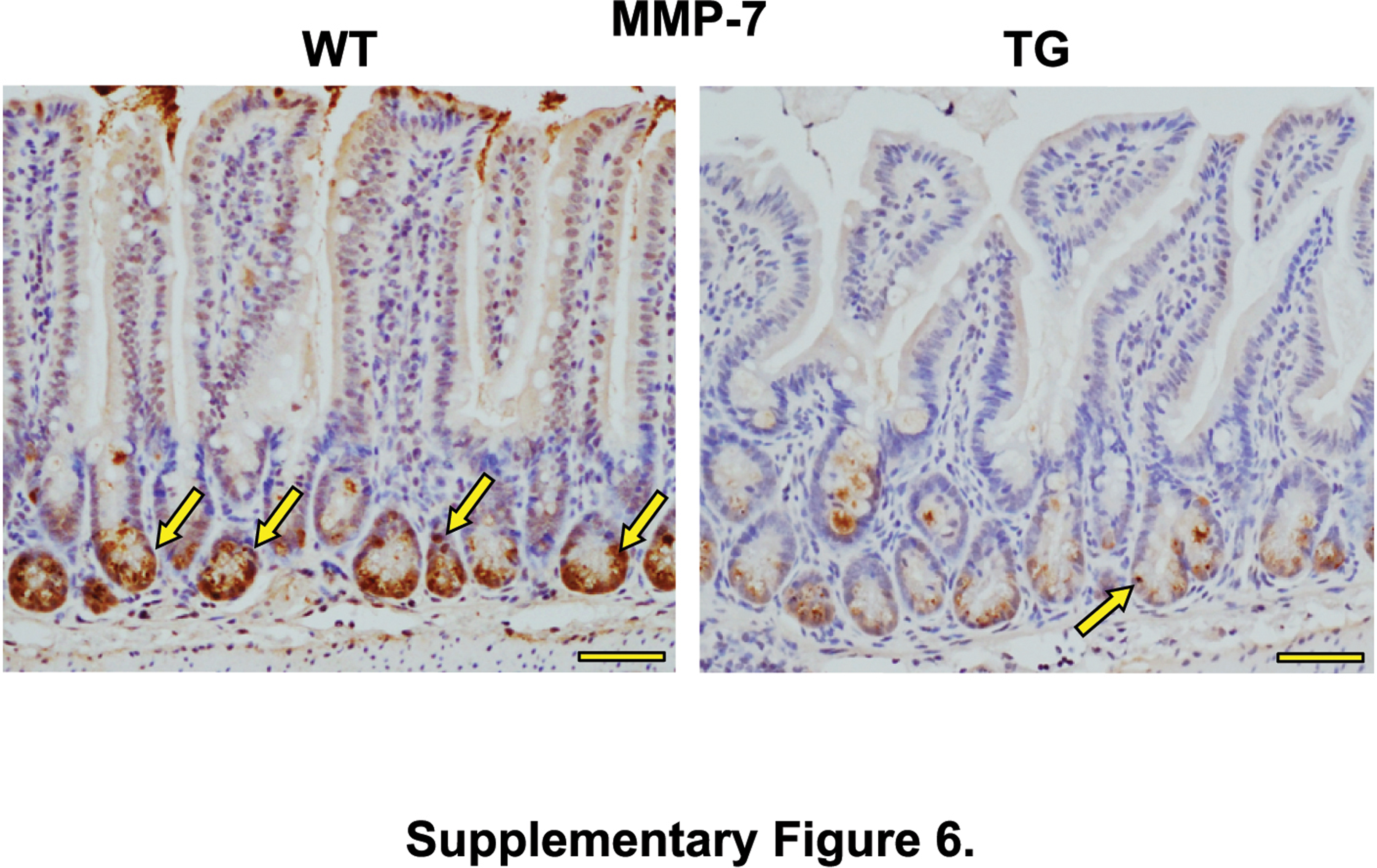

Supplement: Supplementary Figure 6 [file cddis2014588x6.tif]

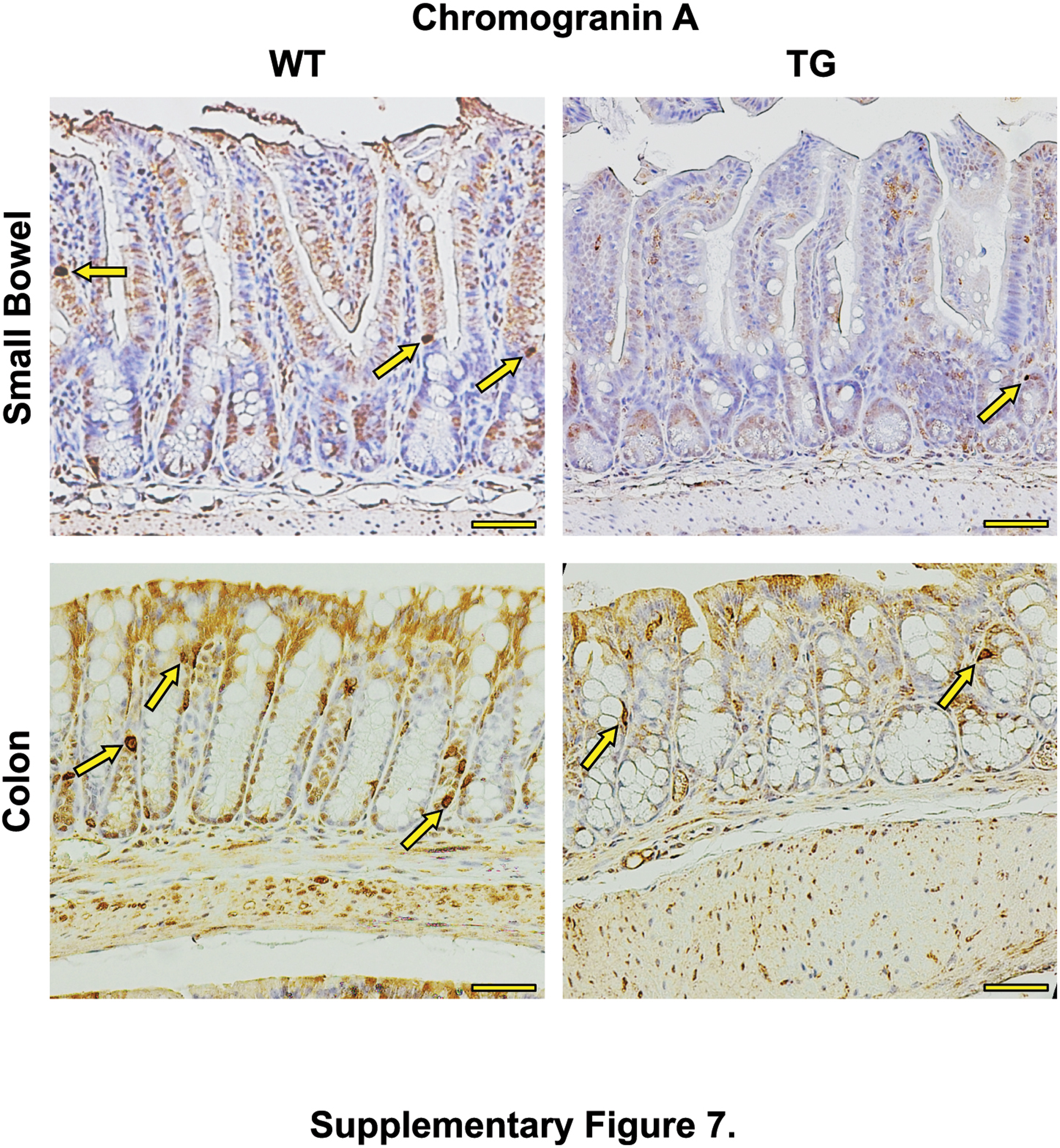

Supplement: Supplementary Figure 7 [file cddis2014588x7.tif]
