# Supplementary material for: TSC2/mTORC1 signaling controls Paneth and goblet cell differentiation in the intestinal epithelium
Source: Cell Death Dis. 2015 Feb 5;6(2):e1631–. doi: 10.1038/cddis.2014.588 (PMC4669793; doi:10.1038/cddis.2014.588)
Supplement: Supplementary Figure Legends [file cddis2014588x8.doc]

**Supplemental Figure 1.** **Identification of TSC2-ΔRG transgene.** PCR identification of TSC2-ΔRG transgene (TG) showed a corresponding band at 280 bp, whereas WT showed a corresponding band at 500 bp.

**Supplemental Figure 2.** **The overall morphology of intestine.** Sections of WT and TG small intestine and colon were examined by histology. Scale bars, 100 μm.

**Supplemental Figure 3.** **Apoptosis is not affected by TSC2 inactivation.** Apoptotic cells

of the small intestine labeled by TUNEL staining. The arrows indicate apoptotic cells. Scale bars, 50 μm.

**Supplemental Figure 4.** **TSC2 inactivation leads to the increased phosphorylation of 4E-BP in intestine.** Intestinal mucosal protein lysates extracted from WT and TG mice were used for Western blot detection of phospho-4E-BP expression.

**Supplemental Figure 5.** **Inactivation of TSC2 increases the proliferation in intestine.** Immunohistochemical staining of the small intestine for Ki67. Scale bars, 50 μm.

**Supplemental Figure 6.** **TSC2 inactivation results in altered Paneth cell differentiation in intestine.** Immunohistochemical staining of the small intestine for MMP-7 showed the decrease in Paneth cells (arrow) in TSC2 mutant TG mice compared to WT mice. Scale bars, 50 μm.

**Supplemental Figure 7.** **TSC2 inactivation results in altered enteroendocrine cell differentiation in intestine.** Immunohistochemical staining of the small intestine and colon for chromogranin A showed the decrease in enteroendocrine cells (arrow) in TSC2 mutant TG mice compared to WT mice. Scale bars, 50 μm.
